# 肺黏膜相关淋巴组织淋巴瘤研究进展

**DOI:** 10.3779/j.issn.1009-3419.2024.106.31

**Published:** 2024-12-20

**Authors:** Shaowei ZHANG

**Affiliations:** 467021 平顶山，联勤保障部队第九八九医院心胸外科（平顶山院区）; Department of Cardiothoracic Surgery, Pingdingshan Medical District, 989^th^ Hospital of Joint Logistics Support Force,Pingdingshan 467021, China

**Keywords:** 黏膜相关淋巴组织淋巴瘤, 肺原发性淋巴瘤, NF-κB信号通路, 利妥昔单抗, 抗生素, Mucosa-associated lymphoid tissue lymphoma, Primary pulmonary lymphoma, NF-κB signal pathway, Rituximab, Anti-bacterial agents

## Abstract

黏膜相关淋巴组织（mucosa-associated lymphoid tissue, MALT）淋巴瘤是肺原发性淋巴瘤中最常见的病理类型，是一种惰性淋巴瘤，其发病与慢性炎症刺激及自身免疫性疾病关系密切，临床表现无特异性且发病率低，常易误诊，明确诊断后最佳治疗措施也存在一定争议。本文综述了肺MALT淋巴瘤的流行病学、发病机制、临床表现、计算机断层扫描（computed tomography, CT）特征、病理诊断、治疗及预后等情况，为进一步认识该病提供参考。

## Abstract (en)

Mucosa-associated lymphoid tissue (MALT) lymphoma is the most common primary pulmonary lymphoma, which is an indolent B-cell lymphoma thought to originate from marginal zone B cells. Its pathophysiology is closely related to chronic antigenic stimulation, regardless of whether the antigens are auto-antigens or of microbial origin. Due to its nonspecific clinical presentation and low prevalence, pulmonary MALT lymphoma is often misdiagnosed. The disease is slow-growing, and different treatments have shown good efficacy, but its optimal treatment is somewhat controversial. This paper reviews the epidemiology, pathogenesis, clinical manifestations, computed tomography (CT) features, pathological diagnosis, treatment and prognosis of pulmonary MALT lymphoma, providing reference for clinicians to further understanding of the disease.

黏膜相关淋巴组织（mucosa-associated lymphoid tissue, MALT）是由Isaacson和Wright在1983年提出，认为其是两种淋巴瘤（地中海淋巴瘤与原发性胃肠道滤泡中心细胞淋巴瘤）共同的组织学发生机制^[1,2]^。次年，两位学者^[3]^报道了4例起源于MALT的淋巴瘤，其中1例发生于肺部。值得注意的是，这并不代表发现了一种新的疾病，更早的时候，类似的肺部病变被Slatzstein称之为“假性淋巴瘤（pseudolymphoma）”，指的是一种相较于发生在其他部位淋巴瘤而言，进展缓慢、预后良好的肿瘤^[4]^。

根据第5版世界卫生组织（World Health Organization, WHO）造血与淋巴组织肿瘤分类，MALT淋巴瘤属于B细胞肿瘤分类中边缘区淋巴瘤（marginal zone lymphoma, MZL）的一种亚型^[5]^（表1），全称为黏膜相关淋巴组织结外边缘区淋巴瘤（extranodal marginal zone lymphoma of mucosa-associated lymphoid tissue, EMZL），起源于后生发中心的记忆B细胞，常发生于正常状况下缺乏MALT的器官，如胃、唾液腺、肺等^[6]^；不同解剖部位的EMZL，其病因、发病机制、遗传学变异及临床表现也存在一定差异^[2,5]^。EMZL是肺原发性淋巴瘤（primary pulmonary lymphoma, PPL）最常见的病理类型，其发病率低，但是占所有PPL的80%左右^[6,7]^，是一种独特的惰性淋巴瘤，预后较好，对包括化疗、放疗在内的多种治疗反应良好。本文旨在对肺部EMZL进行综述。

**表 1:** 黏膜相关淋巴组织淋巴瘤在第5版WHO血液淋巴系统肿瘤分类中的位置

B-cell lymphoid proliferations and lymphomas
Mature B-cell neoplasms
Marginal zone lymphoma (MZL)
Extranodal marginal zone lymphoma of mucosa-associated lymphoid tissue (EMZL)
Primary cutaneous marginal zone lymphoma (PCMZL)
Nodal marginal zone lymphoma (NMZL)
Paediatric marginal zone lymphoma (pNMZL)

WHO: World Health Organization.

## 1 流行病学

肺部EMZL多在60岁左右发病，女性多见（表2）^[8⇓⇓⇓⇓⇓⇓⇓⇓⇓⇓⇓⇓-21]^，也有研究^[6,18,20,22,23]^认为无明显性别倾向。患者的吸烟率与一般人群相当^[8,14,21⇓⇓-24]^。7%-54%的患者确诊时有干燥综合征、类风湿性关节炎、系统性红斑狼疮等自身免疫性疾病^[8,14,18,21⇓-23,25]^（表2）；43%的患者合并单克隆丙种球蛋白血症^[9]^。有研究^[26]^认为近年来肺部EMZL的发病率有所上升。

**表 2:** 近5年关于肺黏膜相关淋巴组织淋巴瘤的临床研究（病例数>20例）一览表

Author	n	Age (yr)	Female	Smoker	Asympto-matic	B symptoms	Autoimmune disease	LDH elevation	Procedures	Staging system	Stage	Therapy	PFS	OS
An^[10]^	48	59.29±10.75	25 (0.52)	11 (0.23)	NA	NA	SS (*n*=3)	NA	TBLB (*n*=12)	Ann Arbor	I (*n*=19)	Surgery (*n*=4)	NA	1-yr 95.74%
									PTNB (*n*=29)		II (*n*=1)	CTx (*n*=20)		
									Surgery (*n*=7)		III (*n*=22)	Surgery+CTx (*n*=8)		
											IV (*n*=6)	CTx+RTx (*n*=4)		
												W&W (*n*=12)		
Shi^[11]^	77	59 (24-85)	45 (0.58)	NA	32 (0.42)	15 (0.19)	5 (0.06)	8 (0.10)	NA	Ann Arbor	I (*n*=14)	Surgery (*n*=19)	5-yr 73.2%	5-yr 95.1%
											II (*n*=3)	CTx (*n*=43)		
											III (*n*=1)	W&W (*n*=15)		
											IV (*n*=59)			
Xu^[12]^	86	56.7±10.6	42 (0.49)	30 (0.35)	48 (0.56)	10 (0.12)	SLE (*n*=1)	NA	TBLB (*n*=5)	Ann Arbor	I/II (*n*=75)	Surgery (*n*=57)	5-yr 75.7%	5-yr 95.0%
							SS (*n*=1)		PTNB (*n*=3)		III/IV (*n*=11)	CTx (*n*=6)	10-yr 35.1%	10-yr 76.8%
							RA (*n*=1)		Surgery (*n*=78)			Surgery+CTx (*n*=18)		
												CTx+RTx (*n*=1)		
												W&W (*n*=4)		
Ning^[13]^	96	59.90±9.88	51 (0.53)	28 (0.29)	NA	NA	0	NA	TBLB (*n*=14)	Modified Ann Arbor	IE/IIE (*n*=96)	Surgery (*n*=48)	mPFS 118 mon	NA
									PTNB (*n*=11)			CTx (*n*=20)		
									Surgery (*n*=71)			Surgery+CTx (*n*=23)		
												CTx+RTx (*n*=5)		
Min^[14]^	42	63 (39-87)	19 (0.45)	16 (0.38)	NA	9 (0.21)	SLE (*n*=1)	11 (0.26)	TBLB (*n*=10)	Modified Ann Arbor	IE (*n*=14)	Surgery (*n*=6)	54.30%	93.90%
							RA (*n*=2)		PTNB (*n*=11)		IIE (*n*=11)	CTx (*n*=34)		
									Surgery (*n*=21)		III (*n*=7)	W&W (*n*=2)		
											IV (*n*=10)			
Wang^[15]^	80	59 (31-80)	43 (0.54)	NA	27 (0.34)	17 (0.21)	NA	NA	TBLB (*n*=33)	Modified Ann Arbor	I (*n*=56)	Surgery (*n*=23)	5-yr 74.6%	5-yr 87.1%
									PTNB (*n*=14)		II (*n*=14)	CTx/R/R-CTx (*n*=31)		
									Surgery (*n*=33)		III (*n*=3)	Surgery+CTx (*n*=6)		
											IV (*n*=7)	W&W (*n*=17)		
												Other^#^ (*n*=3)		
Xu^[16]^	32	59.2±10.0	16 (0.50)	7 (0.22)	22 (0.69)	4 (12.5)	NA	NA	Surgery	Modified Ann Arbor	I (*n*=24)	Surgery	NA	mOS 96.4 mon
											II (*n*=8)			
Yan^[17]^	28	55 (18-85)	11 (0.39)	NA	NA	5	NA	NA	TBLB (*n*=4)	Ann Arbor	IE	R/R-CTx (*n*=14)	mTTF*	mOS*
									PTNB (*n*=9)			W&W (*n*=14)	59 mon	76 mon
									Surgery (*n*=15)				29 mon	78 mon
Husnain^[18]^	40	58.5 (17-80)	18 (0.45)	12 (0.30)	NA	9 (22.5)	5 (0.13)	13 (32.5)	NA	Ann Arbor	I/II (*n*=13)	Surgery (*n*=4)	mPFS 7.5 yr	mOS 15.7 yr
											III/IV (*n*=27)	CTx (*n*=26)	10-yr 32.9%	10-yr 70.2%
												Surgery+CTx (*n*=1)		
												RTx only (*n*=3)		
												CTx+RTx (*n*=4)		
												W&W (*n*=2)		
Joffe^[19]^	127	66 (IQR,	78 (0.63)	75 (0.61)	NA	3 (0.02)	26 (0.21)	9 (0.07)	NA	NA	NA	Surgery (*n*=51)	6-yr EFS 69%	6-yr 93%
		54-74)										CTx (*n*=14)	Surgery 74%	Surgery 100%
												W&W (*n*=58)	CTx 62%	CTx 76%
													W&W 65%	W&W 91%
Kwak^[20]^	67	55 (28-80)	31 (0.46)	NA	40 (0.60)	NA	NA	45 (0.67)	NA	NA	I/II (*n*=30)	Surgery (*n*=16)	10-yr 65.3%	10-yr 83.2%
											III/IV (*n*=37)	CTx (*n*=37)		
												RTx (*n*=1)		
												Surgery+CTx (*n*=3)		
												W&W (*n*=10)		
Gao^[21]^	71	58 (30-80)	35(0.49)	12 (0.17)	44 (0.62)	7 (0.10)	9 (0.13)	9 (0.13)	NA	Modified	I/II (*n*=56)	Surgery (*n*=31)	5-yr 73.6%	5-yr 91.2%
										Ann Arbor	III/IV (*n*=15)	R-CTx (*n*=13)	10-yr 52.8%	10-yr 75.5%
												RTx (*n*=11)		
												W&W (*n*=16)		

Surgery+RTx (*n*=1), RTx (*n*=1), supportive treatment (*n*=1); *The mTTF in the W&W cohort and R/R-Chemo cohort was 29 and 59 months, and the mOS was 78 and 76 months, respectively. IQR: inter-quartile range; LDH: lactate dehydrogenase; PFS: progression-free survival; OS: overall survival; SS: Sjogren^’^s syndrome; TBLB: transbronchial lung biopsy; PTNB: percutaneous transthoracic needle biopsy; CTx: chemotherapy; RTx: radiotherapy; W&W: watch-and-wait; SLE: systemic lupus erythematosus; RA: rheumatoid arthritis; mTTF: median time to treatment failure; EFS: event-free survival; NA: not available.

## 2 发病机制

肺部EMZL的确切病因并不明确。目前认为，EMZL的发生是一个多步骤的过程^[27]^。正常肺中并未发现有支气管相关淋巴组织（bronchus-associated lymphoid tissue, BALT），最早是在胎儿和新生儿受不明性质肺部感染后，在其肺部发现有BALT；BALT也可在滤泡性细支气管炎中出现，并发现其与包括干燥综合征在内的自身免疫性疾病相关^[6]^。在某些病原体或自身抗原的持续刺激下，会使原本缺乏MALT的部位产生一个生发中心，也称为“获得性淋巴组织”。受到炎症过程引起的免疫反应的严重影响，BALT发生肿瘤B细胞的演化并出现克隆性扩增^[7,28]^。在EMZL中已经发现了以易位为主的多种细胞遗传学异常，这些易位产生的融合蛋白通过激活核因子-κB（nuclear factor-kappa B, NF-κB）信号通路，参与EMZL的发生发展^[27]^。有趣的是，EMZL几乎都发生在获得性黏膜相关淋巴组织中，生理上富含MALT的组织器官如回肠末端的Peyer斑罕有EMZL发生，这被认为慢性抗原刺激是EMZL发生的潜在原因^[2,6,28]^。有研究认为与吸烟可能有一定关系，但是，多数研究^[8,24]^表明肺部EMZL患者的吸烟率并不高。

持续的慢性感染，证据比较充足的是幽门螺旋杆菌（Helicobacter pylori, Hp）感染与胃部EMZL的密切关系，90%胃部EMZL合并有Hp感染，而且根除Hp后会使得60%-80%的早期胃部EMZL患者获得长期的完全缓解^[2,6]^。目前并未发现有特定的病原体与肺部EMZL存在因果关系，虽然有研究^[6,29]^发现木糖氧化无色杆菌（Achromobacter xylosoxidans）在肺部EMZL患者中检出率显著升高；但随后的研究没能进一步证实两者相关^[30]^。笔者认为，包括慢性感染在内的持续炎症刺激可能是肺部EMZL发生发展的关键因素，但不代表肺部EMZL是由某一种或某几种特定病原体所诱发；因为只有个别病原体如Hp可以在胃部生存，而肺部或支气管则没有那么苛刻的环境限制。研究肺部MALT的产生，可能更有利于阐明肺部EMZL的发生，从而为治疗寻找新的角度。慢性抗原刺激引起的自身免疫性疾病，如系统性红斑狼疮、多发性硬化症，特别是干燥综合征，都是公认的发生肺部EMZL的危险因素^[6,8,9]^。需要注意的是，合并自身免疫性疾病的EMZL患者，对抗生素治疗反应较差^[2]^。

细胞遗传学异常通过影响NF-κB信号通路促进EMZL发生发展，这些遗传改变包括染色体易位和体细胞突变。不同解剖部位EMZL之间的遗传改变存在一定差异。三体型3号染色体和18号染色体常见于EMZL，但无特异性^[5,7]^。在肺部EMZL最常见的细胞遗传学异常是t（11; 18）（q21; q21），而且是EMZL所特有的^[2,28,31]^。40%左右的肺部EMZL病例中可检测到这种易位^[28,31]^，位于11号染色体上的细胞凋亡抑制剂2（apoptosis inhibitor 2, API2）基因与位于18号染色体上的MALT1基因融合，产生融合蛋白API2-MALT1，导致NF-κB通路的激活^[6,7]^。这是一个潜在的治疗靶点，但肺部EMZL中细胞遗传学异常的预后价值尚不明确^[2,6,7]^。肺部EMZL其他的遗传学异常包括TNFAIP3缺失/突变和启动子甲基化、MYD88突变、TBL1XR1突变等，这些异常均可激活NF-κB通路^[5,7]^。

## 3 临床表现

30%-50%的肺部EMZL患者无症状（表2），仅是因影像学异常而意外发现；有症状者多表现为咳嗽、咯血、胸痛、呼吸困难等非特异症状^[8,23,24]^；约20%患者有B症状，包括：（1）排除感染原因的不明原因发热>38 ^o^C，连续3 d以上；（2）盗汗；（3）体重于诊断前半年内下降>10%^[14,23,25]^，如有发热和体重减轻应警惕向侵袭性疾病转化^[6,25]^。部分患者肺部听诊可闻及捻发音^[6]^。由于其临床表现的无特异性，肺部EMZL常被误诊为肺炎而持续使用抗生素^[7]^。

## 4 影像学表现

胸部计算机断层扫描（computed tomography, CT）是肺部EMZL首选检查，但其表现多样、特异性低。很多患者病变累及双肺、存在多个病灶，可表现为实变影、结节、肿块、磨玻璃样，常伴有病变内支气管充气征、支气管扩张以及病变周围磨玻璃密度，少数可伴有胸腔积液^[32⇓-34]^。还有部分发生于大气道，表现为气管内或主支气管内结节状突起^[35]^。常需与肺炎、肺癌等相鉴别。

多发性结节和/或肿块伴支气管充气征，同时表现为病灶周围毛刺、支气管中心分布及“星系征”，可能有助于CT诊断肺部EMZL^[36]^。黄钦熊等^[37]^研究认为，常见的空气支气管征表现为充气支气管达病灶边缘、无不规则狭窄及壁破坏表现；增强扫描可见血管“造影征”，表现为强化的血管穿过病灶，形态正常，走形自然，无扭曲、变形及增粗表现。其病理基础为肺部EMZL肿瘤细胞沿解剖结构血管支气管树生长，对小叶间隔、肺泡壁形成淋巴细胞样浸润，于细支气管黏膜下淋巴细胞样细胞浸润形成淋巴上皮病损，不破坏支气管及血管^[33,36,37]^。加之病变多累及双肺、多个病灶，通常病变动态变化不显著，可作为CT诊断线索。总之，由于缺乏特征性表现仅通过影像学诊断肺部EMZL存在挑战。

## 5 诊断与病理

肺部EMZL的病理诊断所需标本可通过支气管镜活检、CT或超声引导下肺穿刺以及外科手术获得（表2），但是，无论是支气管镜活检，还是CT或超声引导下的肺穿刺活检，都存在难以明确诊断的情况^[12,15]^。如果通过CT引导穿刺或支气管镜活检获得的标本量小而不足以明确诊断时需要进行手术取材^[2]^。有研究^[6]^认为对支气管灌洗液进行检查有很大的应用前景，但并无进一步研究跟进报道。

肉眼上，肺部EMZL病变呈浅褐色至灰白色、均匀，与周围分界清或不清的无包膜肿块，周围组织可有实变；切面质韧、呈纤维状或细颗粒状。气道多不受影响，而脏层胸膜可受累及而形成息肉样多发结节或白棕色增厚斑块^[6,7,38]^。光镜下显示淋巴细胞样肿瘤细胞浸润破坏正常组织结构。EMZL典型“三联征”包括：反应性淋巴滤泡、中心细胞样淋巴细胞弥漫性浸润和淋巴上皮病变^[7]^。但这些特征可能在小活检标本中易被遗漏，因此免疫组化检查是确定诊断的重要手段。EMZL肿瘤细胞是CD5和CD10均阴性的成熟小B细胞，浆细胞样分化较常见。典型表现为B细胞抗原如CD19、CD20、CD22和CD79a阳性；而CD3、CD5、CD10、CD23、BCL-6和Cyclin D1通常阴性表达^[2,7,39]^。Ki-67增殖指数在肿瘤细胞中较低（<10%），但在残余生发中心通常较高^[7,38]^。

大多数肺部EMZL可根据形态学和免疫表型明确诊断；对于通过形态学和免疫组织化学特征难以确诊的病例，可借助聚合酶链式反应（polymerase chain reaction, PCR）、荧光原位杂交技术（fluorescence in situ hybridization, FISH）等分子生物学技术鉴别诊断^[6]^。检测到t（11; 18）（q21; q21）有助于EMZL诊断^[28]^。

## 6 检查检验

明确诊断为肺部EMZL后通常需要进一步明确是否有肺外器官累及以及合并自身免疫性疾病等情况。约半数有肺外器官累及^[6,23]^，如果需要排除多部位受累，通常需要进行胃镜和结肠镜检查，必要时应对唾液腺和泪腺进行磁共振成像或超声检查评估^[2]^。其他黏膜部位必须仅在有症状的患者中进行评估，这些部位包括眼、耳、鼻和咽喉。骨髓活检不是必需的，但部分患者有骨髓转移^[6,23]^，抗肿瘤治疗前建议进行骨髓活检^[2]^。少部分患者可出现乳酸脱氢酶（lactate dehydrogenase, LDH）水平升高，明显升高的比例较低^[14,23,24]^（表2）。合并自身免疫性疾病可出现相应的自身抗体，确诊肺部EMZL后可选择性进行此类检查。

## 7 临床分期

EMZL最佳分期系统存在争议^[2]^，多数肺部EMZL研究采用Ann Arbor-Cotswolds分期（modified Ann Arbor classification）或Ann Arbor分期（表2）；已有研究^[40]^证实肿瘤原发灶-淋巴结-远处转移（tumor-node-metastasis, TNM）分期可能更适用于眼附属器EMZL，但缺乏肺部EMZL最佳分期系统的研究。有研究^[11,18,21]^发现I/II期与III/IV期患者的生存率无统计学差异，这一方面可能与样本量小有关，另一方面也显示出现有的分期系统对患者预后的预测价值有限，需要开发专门的分期系统或预后模型。

## 8 自然病程

已经有多个病例报道^[41,42]^肺部EMZL在经过活检明确病理诊断后发生自发性的完全消退或部分消退，研究^[41]^认为其可能的机制是对肿瘤的侵入性活检导致身体免疫系统激活而痊愈。鉴于肺部MALT淋巴瘤发病率低，肿瘤消退发生概率可能并不低，提示医生在制定治疗方案时应考虑到该情况。

部分患者会转化为弥漫大B细胞淋巴瘤（diffuse large B-cell lymphoma, DLBCL）^[43]^，一项纳入467例EMZL患者的研究提示，有37例（7.9%）患者组织学类型会转变为DLBCL，值得注意的是，研究中包含37例肺部EMZL，并未观察到该现象。然而，另外有两项研究提示，发生DLBCL的转变率在结外EMZL为2.2%（22/990）^[44]^、非胃原发的结外EMZL为2.7%（20/746）^[45]^。转变为DLBCL与较高的LDH水平、分期较晚、原发部位等因素相关^[43⇓-45]^。发生组织学类型转变的患者预后较差^[44,45]^。

## 9 治疗

迄今为止还没有经随机对照试验（randomized controlled trial, RCT）确定肺部EMZL最佳治疗方法的报道。而且，囿于其低患病率和惰性的临床过程，在可预见的将来进行RCT来比较不同治疗方式的优劣是不切实际的^[22,25]^。目前常用的治疗方法包括手术、放疗、化疗、免疫治疗以及积极观察，这取决于患者的具体情况^[6]^。国内诊疗规范指出，对于非胃原发EMZL，I和II期首选累及部位照射（involved site radiotherapy, ISRT）或手术，因治疗可能产生严重并发症者也可观察等待或单药利妥昔单抗治疗；III和IV期参考III/IV期滤泡性淋巴瘤的治疗^[46,47]^。而2024年更新的美国国立综合癌症网络（National Comprehensive Cancer Network, NCCN）B细胞淋巴瘤指南推荐，对于非胃原发EMZL，IE和IIE期首选ISRT，手术切除对于发生在特定部位（肺、乳腺等）的病变可作考虑。

对肺部EMZL进行手术切除是有争议的，对于局限于肺部的病变，手术切除预后很好^[14,22,25,48]^；但也有研究^[13,15,32]^认为，手术并不一定带来生存获益。这可能在于两个方面：一是样本量不足而显示不出统计学差异，这个因素通过扩大样本量可以解决；二是必须要考虑的领先时间偏倚，解决这个因素，除了扩大样本量之外，还需要严谨的研究设计方案。如果采取手术，手术切除范围尚无定论，主要根据病灶大小及位置而定；是否需要进行淋巴结清扫，目前缺乏相关研究。至少，在已经明确诊断的情况下，手术并非是唯一可以获得良好治疗效果的手段。总体上，手术在肺部EMZL的诊断和治疗中发挥着重要作用，但手术是否带来生存获益仍需要进一步研究。

放疗在诊疗指南与临床研究中的地位差异较大：国内诊疗规范和NCCN指南均推荐ISRT作为IE和IIE期非胃原发EMZL的首选方案；但是关于肺部EMZL放疗的研究较少，且样本量较低^[49]^，涉及较大宗病例的临床研究中单纯放疗较少，多联合化疗应用。放疗与手术均属于局部治疗手段，可获得良好的疾病控制效果，且治疗后生活质量较高^[2,21,22,49]^。总体上肺部EMZL对放疗比较敏感，放疗或单药化疗与手术相比，放疗的并发症发生率似乎更低，更适用于气管或主支气管病变，放疗后可达到完全缓解^[35]^。

与放疗的情况不同，虽然在NCCN指南中对于非胃原发EMZL进行化疗仅推荐用于有症状、胃肠道出血、肿瘤负荷重或病情进展等情况发生的全身性复发患者，但是关于肺部EMZL的化疗相对来说研究更多（表2）。总体上，肺部EMZL对多种化疗方案敏感^[14,50,51]^。有研究^[32,50]^认为化疗可以考虑作为肺部EMZL的一线治疗进行选择；Oh等^[32]^认为相对于手术，化疗的并发症发生率更低、对肺功能影响更小。值得注意的是，对于进展期肺部EMZL，有研究^[6,23]^认为多药化疗方案并不优于单药化疗；与环磷酰胺或蒽环类药物相比，苯丁酸氮芥可能获得更好的无进展生存期（progression-free survival, PFS）^[23]^，但是，近年来的研究^[6,22]^中化疗多联合利妥昔单抗治疗肺部EMZL；对于非胃原发EMZL适用化疗的情形，NCCN指南中推荐的一线化疗方案包括三种首选方案均联合利妥昔单抗，分别是：苯达莫司汀+利妥昔单抗；CHOP（环磷酰胺、阿霉素、长春新碱、泼尼松）+利妥昔单抗；CVP（环磷酰胺、长春新碱、泼尼松）+利妥昔单抗。双肺病变、术后复发或晚期患者适用于包括化疗在内的系统性治疗。

利妥昔单抗多与化疗联合治疗，也可单药治疗，总体反应率较高，部分患者可达到完全缓解^[6,52]^。42例肺部EMZL在内的IELSG-19随机临床试验^[53]^最终结果显示，相较于利妥昔单抗和苯丁酸氮芥单药治疗，利妥昔单抗联合苯丁酸氮芥治疗EMZL拥有更高的无事件生存期（event-free survival, EFS）和PFS，然而，EFS和PFS的改善并没有转化为更长的总生存期（overall survival, OS）；值得注意的是，肺部EMZL相较于其他部位EMZL的EFS较差，因研究中提供信息有限，所以无法判定肺部EMZL治疗反应是否与总体情况一致。Okamura等^[54]^对8例肺部EMZL患者进行了利妥昔单抗单药治疗，发现耐受性良好，所有患者在随访期间（中位时间64个月）均存活，认为利妥昔单抗单药治疗可作为肺部EMZL患者的一线治疗。对于非胃原发EMZL适用化疗的情形，NCCN指南中提到利妥昔单抗单药治疗可作为EMZL一线化疗方案中的次选方案。

对于无症状、身体状况差的患者，可不采取治疗措施而待疾病进展或出现症状后再进行治疗；很多研究^[15,23,52]^中都有一定比例积极观察而不采取治疗措施的患者。一项旨在比较积极观察与利妥昔单药或联合化疗治疗IE期原发性肺部EMZL疗效的多中心研究^[17]^发现，观察组和立即治疗组的中位OS分别为78和76个月，差异无统计学意义（*P*=0.696）。在发现疾病进展后接受治疗同样可以达到完全缓解^[14]^。因此，对于肺部EMZL这种恶性程度低、病程进展慢、对治疗敏感的疾病，需要积极考虑“治症而非治病”的治疗策略。

大多数MZL患者在一线治疗后会出现复发，复发后并无明确二线药物可供选择。有报道^[55]^依鲁替尼（一种Bruton酪氨酸激酶抑制剂）单药治疗曾接受利妥昔单抗治疗的复发/难治性MZL具有长期安全性和有效性。是否适用于复发的肺部EMZL患者可能尚需进一步研究。

虽然EMZL的发病被认为与慢性感染有关，但并未发现特定病原体与肺部EMZL有明确因果关系。因此，抗生素治疗不是主要治疗方式。Ferreri等^[56]^报道了一项II期临床试验，发现大剂量克拉霉素对复发或难治性EMZL患者安全有效，其中有肺部EMZL患者显示出对克拉霉素的良好活性。Lagler等^[57]^报道的另一项II期临床试验，发现长期口服阿奇霉素治疗EMZL同样显示出一定的抗淋巴瘤活性，其中包含2例肺部EMZL患者，1例部分缓解，1例病情稳定。基于肺部EMZL对抗生素的治疗反应，实际上肺部EMZL发病率可能更高。

## 10 预后及影响因素

由于其恶性程度低、病情进展缓慢，总体预后很好，各研究中肺部EMZL患者5和10年的疾病特异性生存率分别约为90%和70%，中位OS>10年^[6,9,54]^，患者最终死亡归因于EMZL的比例并不高^[23]^。对于预后的影响因素，不同研究之间的结论并不一致。与生存相关的预后因素有年龄、体力状况评分、疾病分期、系统性症状的存在、是否合并自身免疫性疾病、肺部病变的数量和位置、副蛋白血症的存在以及治疗类型^[6,15,23]^。肿瘤数量与PFS相关，年龄是影响肺部EMZL患者预后的唯一独立危险因素^[15]^。有趣的是，有研究^[23]^认为肺外病灶的存在并不影响OS和PFS。

综上，肺部EMZL是一种惰性淋巴瘤，其发病与慢性炎症刺激及自身免疫性疾病关系密切；临床表现无特异，CT表现多样，诊断依赖形态学和免疫组织化学；病情进展缓慢，治疗手段多样，治疗反应良好，总体预后很好。
